# Comparison of visual outcomes, spectacles dependence and patient satisfaction of multifocal and accommodative intraocular lenses: innovative perspectives for maximal refractive-oriented cataract surgery

**DOI:** 10.1186/s12886-017-0411-9

**Published:** 2017-02-14

**Authors:** Raffaele Nuzzi, Federico Tridico

**Affiliations:** 1 Institute of Ophthalmology, Department of Surgical Sciences, University of Turin, Via Juvarra 19, 10100 Turin, Italy; 2 Unit of Ophthalmology, San Luigi Gonzaga University Hospital, University of Turin, Regione Gonzole 10, Orbassano, 10043 Turin, Italy

**Keywords:** Cataract surgery, Refractive surgery, Multifocal IOLs, Accommodative IOLs

## Abstract

**Background:**

The aim of this study was to evaluate visual outcomes for different working distances (far, 60 cm and 33 cm) and impact on vision quality of multifocal IOLs AcrySof ResTOR SN6AD1 and SN6AD3 (Alcon, Inc., Fort Worth, Texas, USA) as well as REVIEW FIL611PV multifocal and OPTOFLEX FIL618 accommodative IOLs (Soleko, Ltd., Rome, Italy) in patients undergoing bilateral phacoemulsification.

**Methods:**

In this observational prospective study 63 patients undergoing binocular cataract surgery were divided into four groups for implantation of one of the IOLs under evaluation. Visual outcomes were evaluated at 1 day, 7 days, 1 month, 3 months and 6 months after surgery. Patients’ satisfaction and spectacle independence were evaluated with questionnaires administered at the 6-months follow-up.

**Results:**

Improvements in visual acuity for the three working distances were statistically significant in all cases compared to the preoperative status, especially after binocular implantation. The AcrySof ReSTOR SN6AD1 multifocal IOL provided the best visual acuity results and tolerability for all working distances. While performing worse than SN6AD1, FIL611PV and FIL618 provided better uncorrected visual acuity and spectacles independence for intermediate/close-up and far distances respectively, in comparison with the SN6AD3 group.

**Conclusions:**

SN6AD1 was confirmed the best choice for all working distances. However, FIL611PV IOL may represent a valid and more cost-effective alternative, especially if surgeons intend to prioritize spectacle independence and patient autonomy at intermediate and close-up distances, in accordance to specific needs and requests.

**Trial registration:**

Trial retrospectively registered in ISRCTN Registry on 02/02/2017. TRN: ISRCTN14145737.

## Background

As of today, surgical correction of post-phacoemulsification aphakia with intraocular lenses (IOLs) implant is the most physiological and widely used procedure.

Currently, it is usual to implant, in most cases, monofocal IOLs, which generally require the use of glasses for carrying out tasks at intermediate and close up distance [1, 2].

The more rarely employed multifocal IOLs have been recently developed with the goal of restoring near distance vision, in addition to lens transparency. These lenses create two or more images of the same object falling on different focal points [3, 4]. Habit and cerebral learning make patients select images positioned closer to the retina, neglecting other kinds of images [5, 6]. Multifocal lenses provide a certain kind of multifocal vision by exploiting the lens optical portion constructive design, which maintains its position relatively unchanged when the subject focuses its attention either on close or long distance objects [7–9].

Multifocal IOLs are distinguished between [10–12]:

- optical refractive multifocal IOLs: the lens front surface has two or more concentric rings with different curvature radius, creating bi- or multi-focal vision. Performance of lenses with refractive optics depend on pupil diameter, since the percentage of light that passes through the different optical zones varies depending on pupillary dynamics;
- optical diffractive multifocal IOLs. On the front/back surface of the lens there are numerous concentric rings, separated from each other by a step of around 2 μm in height. These steps diffract light, allowing the creation of two or more foci regardless of pupil size.

Accommodative IOLs are another premium IOL type which are conceived in order to preserve the accommodation capability of the ocular dioptric system, which is lost after cataract extraction. These are lenses with dynamic features, which take advantage of ciliary muscle residual activity, providing, during its contraction, an anterior movement of the IOL optical plate in the posterior chamber. This anterior displacement of lens optic plate provides an increase of its optical power, which triggers a pseudo-accommodation phenomenon, reducing the need for additional spectacles [13, 14]. However, at the present time, there is evidence that patients receiving accommodative IOLs had small gain in near visual acuity and seem to suffer more from posterior capsular opacification, impairing significantly the IOL intended action and distance visual acuity [15, 16].

Advantages of accommodative lenses, in comparison with multifocal IOLs, should comprehend smaller presence of adverse events such as halos, blurs and glows [17–19], frequently associated with both diffractive and refractive multifocal IOLs. The optical plate of an accommodative IOL usually maintains the same power in every point, with absence of discontinuity/transition areas, responsible of the issues often associated with multifocal lenses utilization [5, 6, 12, 17–19]. Still, other accommodative IOL models have zones with different optical properties. For instance, Crystalens HD models (produced by Bausch & Lomb, Inc.) have a 1.5 mm diameter modification in the center, resulting in increased negative spherical aberration with a gradual increase in positive spherical aberration, moving toward the periphery of the lens [20, 21].

The aim of this observational, prospective, single-site study, is to evaluate and compare visual performance and quality outcomes at different working distances – far, intermediate (60 cm) and near (33 cm) - of three different multifocal IOLs - AcrySof ReSTOR SN6AD1, SN6AD3 (Alcon, Inc, Fort Worth, Texas, USA) and REVIEW FIL611PV (Soleko Ltd, Rome, Italy) – and the accommodative IOL OPTOFLEX FIL 618 (Soleko Ltd, Rome, Italy).

## Methods

Candidates for bilateral cataract extraction surgery were recruited. Each subject underwent complete ophthalmologic evaluation in the 90 days before procedure (as part of a standard preoperative practice), with:

- collection of personal data.
- ocular examination of at the slit lamp, comprehensive of fundus examination after pharmacological mydriasis.
- acquisition of keratometric values with Javal ophthalmometry, performed always by the same operator (values were obtained from the mean of three consecutive measurements).
- acquisition of corneal topography data with Oculus Pentacam.
- intraocular pressure measurement with Goldmann applanation tonometry.
- execution of contact eye biometry with ultrasound and optical method (no significant differences between these two procedures; *p* > 0.1).
- manifest refraction measurement.
- uncorrected and best-corrected visual acuity examination for far (UCDVA and BCDVA, with the ETDRS charts), intermediate (60 cm, UCIVA and BCIVA) and close-up distances (33 cm, UCNVA and BCNVA, with Jaeger tables).
- measurement of the mesopic pupil size with Slit lamp-based Cobalt Blue Light method (SCBL) [22] in ambient lighting conditions described by Chaglasian (mesopic pupillary diameter value was obtained with the mean of three consecutive measurements) [23].
- orthoptic assessment; evaluation of ocular dominance for far and close-up distances.

Inclusion criteria were Age > 50 years, bilateral cataract and maximum regular corneal astigmatism of 1.0 D. We excluded patient with optical means opacities (different from cataract), age-related macular degeneration, previous history of ocular surgery, irregular corneal astigmatism, amblyopy, concurrent neuro-muscular diseases (cerebral ictus, myasthenia, etc.), uncontrolled open/close-angle glaucoma, severe ocular complications related to diabetes (retinopathy, macular edema, vitreal hemorrhage, etc.) and with intra/postoperative complications. Patient with pupil diameter ≤ 5,2 mm in mesopic lighting conditions were also excluded from the study.

We recruited a total of 63 subjects which were assigned to four different groups undergoing bilateral implantation of one of the IOLs evaluated in this study. Subjects’ eligibility was assessed when patients affected by bilateral cataract attended to a visit at our center during the study duration. Eligible patients were then recruited at the end of preoperative evaluation and allocated in different groups. In order to assign patients to a group, we performed a block randomization (block size of 8), randomizing 8 patients at a time (allocating 2 patients per group for every block selection). 16 patients (32 eyes) were assigned to the SN6AD1 group (a single-piece diffractive multifocal IOL provided with an additional power of +3.0 D, nine diffractive steps and a central optic zone for intermediate distances), 16 to the SN6AD3 group (a single-piece diffractive multifocal IOL with additional power of +4.0 D, 12 diffractive steps and central optic zone for intermediate distances) [24], 16 to the FIL611PV group (single-piece refractive multifocal IOL with four haptics, an additional power of +3.75 D and a central optic zone suited for near distances) [25] and 15 to the accommodative FIL618 group (a single-piece accommodative IOL provided with an annular peripheral haptic and three helical loops, which are conceived to allow movements of its optical zone in accordance with capsular and vitreal modifications, providing an estimated accommodative potential of +2 D) [26]. In patients implanted with FIL618 accommodative lenses, correlations between diameter of the performed capsulorhexis, ocular axial length, pre-operative refractive defect and additional correction requested at 6-months follow-up were also evaluated. In patients with hyperopic visual defect larger capsulorhexis (ideally 6 mm) were performed, in comparison with normally sighted/myopic patients (ideally 5.5 mm). This increased size of the capsulorhexis was supposed to provide greater freedom of movement to the IOL optic plate, along the anteroposterior axis, allowing the patient to get a greater accommodative ability in intermediate range vision [27, 28]. Detailed characteristics of the population under study are reported in Table 1. Our institutional Ethics Committee has been consulted prior to initiating the study for approval. Informed consent was obtained from each recruited subject prior intervention.

**Table 1:** Summary of study population

	SN6AD1	SN6AD3	FIL611PV	FIL618
TOTAL SUBJECTS	16	16	16	15
MALE	10	7	7	9
FEMALE	6	9	9	6
MEAN AGE	72.9	71.8	70.87	70.8

Each patient received preoperative prophylactic treatment consisting of moxifloxacin eye drops 5 mg/ml, 1 drop 3 times/day in both eyes for 3 days before the surgery, according to our institute’s protocol. Therefore, each recruited patient underwent bilateral cataract extraction surgery (each eye was operated during different operative session, one 30 days after the other). All procedures were performed by the same single surgeon as described below:

- Application of topical anesthesia with benoxinate 0.4% drops, followed by disinfection with povidone-iodine.
- Temporal corneal incision of 2.2 mm. Introduction of viscoelastic in anterior chamber
- Execution of continuous circular capsulorhexis. Rhexis diameter measurements in the FIL618 were performed with a mm. caliper used on the excised anterior capsule (previously colored with Trypan blue 0.1%) which was placed on the anterior corneal surface.
- Cataract extraction with phacoemulsification technique.
- IOL introduction in the capsular bag.

Patients were asked to show up at follow-up ophthalmological visits one day (t1), 7 days (t2), 1 month (t3), 3 months (t4) and 6 months after surgery (t5). In the occasion of the last three control visits, the following assessments were performed:

- examination of manifest refraction, uncorrected and best-corrected visual acuity for far, intermediate and close-up distances.
- intraocular pressure measurement.
- anterior segment and fundus examinations with slit lamp biomicroscopy, after pharmacological mydriasis, if necessary.
- acquisition of keratometric values performed always by the same operator (mean values were obtained after three consecutive measurements).
- acquisition of corneal topography data with Oculus Pentacam with collection of central corneal thickness values, anterior chamber depth and iridocorneal angle width.
- optical coherence tomography (OCT) of the macula.
- measurement of photopic pupil size with slit lamp ocular biomicroscopy (mean value was obtained after three consecutive measurements).
- mesopic pupil size measured at the slit lamp with SCBL method (mean values obtained after three consecutive measurements). In the accommodative IOL group, correlations between capsulorhexis diameter, ocular axial length and optical correction at different distances required before surgery were also evaluated.

During one-month and 6-months follow-up visits, orthoptic assessment and evaluation of ocular dominance for far and close-up distances were also performed. Moreover, during the last three visits patients were given a questionnaire designed to assess their satisfaction, the presence or absence of vision-disturbing elements and spectacle independence in different conditions of daily life. All procedures in this study concerning his conduction and documentation were performed in conformity with the ethical principles set out in the Helsinki Declaration and its revisions. Preoperative status and postoperative outcomes were confronted for each group with T-student test. Visual outcomes, patients’ satisfaction and spectacles independence between groups were confronted using the one-way ANOVA test. Correlations, when taken into considerations, were evaluated using the Pearson correlation coefficients.

## Results

Improvements in visual acuity at different follow-up visits were observed for all the IOLs implanted for all working distances. All differences detected at 6-months were statistically significant in comparison with t0. In the following we disclose detailed reports for each study group, comparing also postoperative visual function and patients’ satisfaction/spectacles independence as well. No significant differences of keratometric values were observed between preoperative status and follow-up visits.

### Visual outcomes

Monocular uncorrected and best-corrected visual acuity comparisons between preoperative status and 6-months follow-up for each working distance in all groups are illustrated in Table 2. A significant improvement in monocular visual acuity was observed for all working distances at the 6-months follow-up in the SN6AD1 group (*p* < 0.05). Both uncorrected and corrected binocular visual acuity were improved at 6-months after surgery. Differences between t0 and t5 were statistically significant (*p* < 0.05).

**Table 2:** Preoperative vs. 6-months mean visual acuity for all working distances

	SN6AD1		SN6AD3		FIL611PV		FIL618	
	t0	t5	t0	t5	t0	t5	t0	t5
UCDVA OD	0.61	0.11	0.64	0.16	0.75	0.39	0.66	0.24
UCDVA OS	0.65	0.12	0.69	0.17	0.71	0.32	0.75	0.17
(LogMAR)								
UCIVA OD	9.20	3.23	10.02	9.2	7.57	3	9.2	6.1
UCIVAOS	10.11	3.26	10.12	9.6	6.50	3	12.41	6.66
(Jaeger)								
UCNVA OD	11.90	3.10	13	4.2	8.00	3.53	10	6.8
UCNVA OS	12.20	3	12.5	3.9	7.50	3.56	13.33	7.66
(Jaeger)								
BCDVA OD	0.32	0.03	0.35	0.08	0.30	0.10	0.27	0.04
BCDVA OS	0.30	0.04	0.4	0.08	0.37	0.07	0.37	0.07
(LogMAR)								
BCIVA OD	4.00	3	4.1	5.5	3,67	3	4	3.4
BCIVA OS	4.00	3	4.11	5.4	3,44	3	4	3
(Jaeger)								
BCNVA OD	4.00	2.87	4	3.60	3,90	3	4.25	3.14
BCNVA OS	4.00	2.97	4	3.15	3,54	3	4	2.89
(Jaeger)								

t0 = preoperative status; t5 = 6-months follow-up

Even the SN6AD3 group’s subjects have shown an improvement in visual acuity for all working distances. All differences were statistically significant (*p* < 0.05).

Improvement in visual acuity for the three working distances after FIL611PV implantation was statistically significant in all cases compared to preoperative status.

No significant changes were observed between monocular and binocular uncorrected visual acuity for intermediate distance (UCIVA) (*p* = 0.164). There was a positive trend towards improvement of corrected binocular vision for far distances, if compared with monocular vision. However, this difference is not statistically significant (*P* > 0.05). Comparisons were performed only with BCDVA, because for intermediate and close-up distances no patient required the use of lenses in binocular vision.

Regarding monocular vision performances for near distance, there was a slightly improvement between UCNVA and BCNVA, albeit statistically significant (*p* < 0.0001). No differences were noted between binocular UCNVA and BCNVA.

Even in the FIL618 group improvements in visual acuity for the three working distances were statistically significant in all cases compared to the preoperative status. Binocular uncorrected visual acuity was better than monocular visual acuity and this difference was statistically significant in all cases (*p* < 0.05). Additional correction request with spectacles, in order to obtain the best binocular visual acuity was lower for both near and intermediate distance. This difference was statistically significant in both cases (*p* ≤ 0,001). The additional correction required to obtain the best visual acuity for near and intermediate distance appeared lower in binocular vision than monocular vision, and this decrease was in both cases statistically significant (*p* < 0.05).

In this group, we also evaluated the following correlation with Pearson’s coefficients:

- rhexis diameter measured at the slit lamp at six-months follow-up (t5) (mean 5.73 mm, SD 0.45) and ocular axial lenght (23.23 mm, SD 0.82): the correlation was statistically significant (correlation coefficient of −0.417, *p* = 0.034).
- capsular rhexis diameter at six months (t5) and distance correction required before surgery (spherical equivalent): the correlation was statistically significant (correlation coefficient of 0.740, *p* = 0.0001);
- capsular rhexis diameter at t5 and additional correction requested by the patient to achieve the best visual acuity for intermediate (BCIVA) and close-up distance (BCNVA): no statistically significant correlation has emerged (correlation coefficient of 0.124, *p* = 0.791, in the first case; correlation coefficient of - 0.154, *p* = 0.741, in the second case);
- ocular axial length and additional correction requested by the patient to achieve the best visual acuity for intermediate (BCIVA) and close-up (BCNVA) distance: no statistically significant correlation has emerged (correlation coefficient - 0.32, *p* = 0.93, in the first case; correlation coefficient 0.161, *p* = 0.617, in the second case);
- correction required for far distance before surgery (spherical equivalent) and additional correction requested by the patient to gain best visual acuity for intermediate (BCIVA) and close-up (BCNVA) distances: no statistically significant correlation has emerged (correlation coefficient 0, 33, *p* = 0.951, in the first case; correlation coefficient of −0.409, *p* = 0.314, in the second case).

### Group comparisons

Visual outcomes of each group at 6-months follow-up were confronted. Among all groups considered, SN6AD1 provided the highest monocular uncorrected visual acuity for all distances (*p* < 0.05 for all working distances) and the best monocular BCVA for long and intermediate range, as well. Patients in this group referred also a better binocular distant visual acuity, but overall differences were significant only in case of UCDVA (*p* < 0.01; while for BCDVA *p* = 0.48).

However, the SN6AD3 group featured a monocular BCNVA similar to the SN6AD1 group (95% CI −0.001 – 0.001; *p* = 1) and better than the one observed in the FIL611PV group (95% CI 1.97 – 1.99; *p* < 0.001). Even so, SN6AD3 provided a lower monocular UCIVA in comparison with FIL611PV and FIL618 IOLs. (95% CI 5.86 – 6.36 and *p* < 0.001 if compared with FIL611PV; 95% CI 1.59 – 4.45 and *p* < 0.001 if compared with FIL618). No significant differences in UCDVA were observed between SN6AD3 and SN6AD1 group (95% CI −0.12 – 0.02; *p* = 0.17).

Monocular UCIVA values were higher in the FIL611PV group, if compared with the FIL618 one (95% CI 1.63 – 4.54; *p* < 0.0001), nevertheless these differences were nullified if an additional correction was applied in patients who received FIL618 (95% CI −0.24 – 0.42; *p* = 0.59). For FIL611PV IOLs, BCIVA was not evaluated because no patients in the group required additional correction in binocular vision.

In this study FIL611PV IOLs provided also the best binocular uncorrected intermediate visual acuity. Differences in binocular UCIVA between SN6AD1 and FIL611PV group were little (mean values 1.05 and 1 Jaeger, respectively), albeit statistically significant (95% CI 0.04 – 0.06; *p* < 0.0001).

Subjects who received FIL611PV IOLs had also better uncorrected near visual acuity in comparison with the SN6AD3 group (*p* < 0.0001 for OD; *p* = 0.29 for OS and *p* < 0.0001 if data from both eyes is considered, with 95% CI 0.32 – 0.68). Binocular mean UCNVA was higher in FIL611PV patients in comparison with other IOLs (*p* < 0.0001), with performances similar to SN6AD1 IOL (95% CI −0.05 – 0.25; *p* = 0.19). Binocular UCDVA was slightly worse in the FIL611PV group (95% CI 0.07 – 0.35; *p* < 0.01, if compared with SN6AD1 group).

FIL618 IOLs provided better monocular BCDVA in comparison with SN6AD3 group, but this difference wasn’t statistically significant (95% CI −0.01 – 0.05; *p* = 0.23 with data from OO taken into consideration). However, distance corrected visual acuity was significantly better in the FIL618 group in comparison with the FIL611PV one (95% CI 0.05 - 0.11; *p* < 0.001). However, patients who received OPTOFLEX accommodative IOLs reported a higher BCIVA than patients receiving SN6AD3 IOLs (95% CI 2.09 – 2.51 and *p* < 0.0001).

### Visual disturbances, performance in everyday tasks and spectacles dependence

Patients’ satisfaction data regarding different aspects were collected through questionnaires administered to all subjects under study. Table 3 and 4 show in detail responses given about subjective visual disturbances, frequency of glasses/contact lenses application and overall visual quality. Visual disturbances appeared less frequently in SN6AD1 patients in comparison with SN6AD3, with particular regards to glare and halos (p-values in these cases were 0.03 and 0.003 respectively). No significant differences were found regarding glare incidence between the SN6AD3 and FIL611PV group, however halos and visual difficulties for far distances were more frequent in the FIL611PV. Patients receiving the accommodative IOL referred more frequent visual disturbances with the exception of halos and vision difficulties at long distances, which occurred more in the FIL611PV group (*p* < 0.001 in all cases).

**Table 3:** Comparison of optical correction utilization rate and overall visual quality satisfaction, as referred by patients

	SN6AD1	SN6AD3	FIL611PV	FIL618
How often do you use lenses for far distances?	8,00 (3.57)	7.06 (0.29)	7.83 (1.06)	8,86 (0,74)
How often do you use lenses for intermediate distances?	8.12 (0.28)	7.24 (0.20)	8.41 (0.77)	9,14 (0,59)
How often do you use lenses for close-up distances	8.12 (0.27)	7.24 (0.18)	8 (1.1)	3,86 (1,58)
Global visual quality satisfaction	6.94 (0.25)	6.71 (0.2)	7 (0.57)	7,11 (1,19)

Patients’ answers to questonnaries (0 = always/completely unsatisfied – 10 = never/completely satisfied). Mean values (SD)

**Table 4:** Comparison of subjective visual disturbances frequency, as referred by patients

	SN6AD1	SN6AD3	FIL611PV	FIL618
Glare	0,41 (0.38)	0,65 (0.18)	0,81 (0.42)	1,71 (0,61)
Night vision	0,41 (0.30)	0,69 (0.23)	1,09 (0.53)	1,86 (0,67)
Colour perception	0,44 (0.33)	0,65 (0.29)	0,45 (0.31)	1,14 (0,46)
Depth perception	0,41 (0.34)	0,65 (0.22)	1,81 (0.58)	1,42 (0,81)
Halos	1,41 (0.30)	1,73 (0.25)	2,54 (0.65)	1,57 (0,71)
Distorted close-up vision	0,44 (0.32)	0,65 (0.31)	0,81 (0.44)	1,57 (0,53)
Distorted vision for far distances	0,41 (0.33)	0,65 (0.26)	2,00 (0.68)	1,00 (0,58)
Obscured close-up vision	0,41 (0.27)	0,65 (0.21)	0,91 (0.51)	1,71 (0,47)
Obscured vision for far distances	0,41 (0.24)	0,61 (0.23)	2,81 (0.61)	0,86 (0,34)
Doubled vision	0,44 (0.31)	0,69 (0.27)	1,36 (0.56)	0,71 (0,71)

Subjective visual difficulties at t5 (0 = no difficulties; 5 = maximal difficulty) Mean values (SD)

At 6-months after surgery all subjects referred a favorable trend towards glasses/contact lenses independence for far distances (*p* = 0.09). However, patients belonging to the FIL618 group reported a higher spectacles dependence for close-up distances (*p* < 0.0001). At intermediate distance the need of additional optical correction was lower in the FIL611PV group if compared to the SN6AD3 sample (95% CI 0.76 – 1.58; *p* < 0.0001).

Table 5 shows a detailed report on subjective visual difficulties experimented by patients while performing different everyday tasks without using spectacles/contact lenses. Finally, ANOVA analysis showed no differences in overall visual satisfaction among patients that received multifocal IOLs in this study. Even so, patients in the FIL618 group reported a positive trend about their overall visual quality (95% CI 0.07 – 0.93; *p* = 0.02 if compared with the SN6AD3 group).

**Table 5:** Comparison of subjective visual difficulties in daily tasks without optical corrections, as referred by patients at 6-months after surgery (0 = no difficulties; 5 = maximal difficulty)

	SN6AD1	SN6AD3	FIL611PV	FIL618
Watching TV or movies	0,13 (0,3)	0,75 (0.4)	1,00 (0,40)	1,29 (0,71)
Play/work in external environment	0,13 (1,77)	0,75 (0.58)	0,45 (0,31)	1,00 (0,58)
Take care of/play with children	0,14 (0,37)	0,86 (0.27)	0,18 (0,18)	0,43 (0,30)
Read the time on the alarm clock	0,13 (0,34)	0,75 (0.40)	0,36 (0,20)	1,00 (0,53)
Clarified vision at wake-up	0,13 (0,32)	0,71 (0.22)	0,55 (0,31)	1,28 (0,52)
Read the time on a wall clock	0,13 (0,33)	0,71 (0.33)	0,64 (0,31)	0,86 (0,55)
Work/having an hobby	0,13 (0,35)	0,8 (0.3)	1,09 (0,41)	0,71 (0,47)
Participate to recreative activities/sports	0,29 (0,34)	1,5 (1.22)	0,73 (0,38)	0,57 (0,37)
Participate to social events	0,15 (0,24)	0,92 (0.64)	0,36 (0,36)	0,71 (0,36)
Read texts/close-up tasks	0,13 (0,24)	0,75 (0.15)	0,55 (0,28)	2,00 (0,65)
Driving at night	0,17 (0,27)	1,00 (0.35)	1,00 (0,50)	2,14 (0,63)
Driving in raining conditions	0,17 (0,2)	1,00 (0.2)	0,90 (0,44)	1,71 (0,57)
Use the computer	0,22 (0,16)	1,09 (0.15)	1,18 (0,50)	1,71 (0,61)
Cook	0,13 (0,15)	0,86 (0.15)	0,27 (0,27)	0,57 (0,43)
Going shopping	0,12 (0,23)	0,71 (0.35)	0,55 (0,39)	0,71 (0,47)
Use a cell phone	0,13 (0,3)	0,75 (0.35)	1,09 (0,44)	1,71 (0,65)
Shaving/make-up	0,13 (0,3)	0,71 (0.58)	0,18 (0,18)	1,14 (0,73)

Mean values (Standard Deviation)

## Discussion

Previous clinical studies evaluated functional optical effects and quality of life after implantation of multifocal IOLs including the ReSTOR IOLs experiencing good vision for both distance and for near [29, 30]. Although patients receiving this IOLs showed improved spectacle independence they however reported a lower intermediate visual acuity and a lower contrast sensitivity than patients implanted with monofocal IOLs [31].

Discomforts have been in part opposed by passing from spherical to aspherical IOL technology allowing the reduction of corneal spherical aberration as reported by Alfonso et al. [8].

It has been reported that ReSTOR IOLs lead to mild discomfort for distant vision, while near visual acuity seems to be better, in terms of quantity and quality. For intermediate visual acuity, ReSTOR lenses with a + 3 D additional, unlike other multifocal lenses, seem to guarantee a lesser discomfort.

Multifocal lenses according Javitt and Steinert [6] also show visual discomfort due to the presence of glare/halos (due to the transition zone between the “diffractive step”, unlike accommodative lenses that use the refractive principle of monofocal lenses).

Finally, in terms of spectacles dependence, it is basically found that patients with higher need of visual aids are those implanted with accommodative lenses; multifocal lenses may require occasional use of the refractive supports in case of low-light or tasks performed at intermediate distances. From last meta-analysis of Cochener et al. [32] it is evident that ReSTOR multifocal IOLs turn out to be the ones with the best results and best independence from visual aids, superior to all other lenses available so far on the market.

In this study, visual acuity improvements at 6-months after surgery have been observed in all groups. Performance in terms of uncorrected visual acuity for the three working distances were improved in case of binocular implantation for all the IOLs used in this study. Compared to monocular visual acuity the gain in binocular vision resulted of about a line at visual acuity chart examination. In fact, as often reported in several studies, bilateral implantation of multifocal intraocular lenses can maximize vision for all distances, and provide other vision-related improvements such as better stereopsis (with better patients’ movements) and overall satisfaction, thus favoring bilateral implantation [33–37].

Concerning the AcrySof ReSTOR IOLs, best corrected visual outcomes for far distances in our study were lower if compared with the existing literature (ranging from 0.03 – 0.1 and 0.01 – 0.14 LogMAR for SN6AD1 and SN6AD3, respectively), while more positive outcomes were observed for intermediate distances (range 0.15 – 0.3 for SN6AD1, 0.2 – 0.4 for SN6AD1 in previous studies). In the AcrySof ReSTOR IOLs groups, outcomes for UCNVA and BCNVA were in accordance with previous studies. If we consider uncorrected near visual acuity SN6AD1 performed better, compared to SN6AD3 model, but these differences were nullified in this study if an additional optical correction was applied.

The available literature regarding FIL611PV and FIL618 IOLs is currently very poor. Results from this study show that, if compared with the SN6AD3 group, patients receiving FIL611PV reported a higher visual acuity for intermediate and near distances, while the FIL618 group showed better intermediate and far visual outcomes. Uncorrected visual acuity for intermediate and near distances was decent in the FIL611PV group, especially in binocular vision conditions. These findings are in accordance with what reported by patients in the satisfaction questionnaires, which show a significant spectacle dependence only for far distances. FIL611PV lenses did not provide high performances in natural vision for distance: This could be largely due to tendency of FIL611PV multifocal lenses to induce myopia, especially in photopic conditions, in the postoperative period, equal to approximately −1.08D in monocular vision conditions, with a non-statistically significant reduction to −0.90 diopters binocularity conditions. Such refractive result is precisely linked to the constructive lens design (which like all multifocal lenses has refractive performance directly dependent on pupillar diameter) [38], with the near optical zone located in the center of the optical plate, which penalizes performances under miosis conditions. These findings appear to be related to the constructional design of the lens and not to the surgical technique that, as is clear from the biometric data detected with Oculus Pentacam corneal tomography, did not significantly influenced corneal astigmatism, albeit modifying, as it was natural to expect, two other variables such as angle size and anterior chamber depth.

FIL618 lenses allow to achieve an almost complete spectacle independence for distance vision, according to manufacturer reports and literature data regarding accommodative lenses, which generally have the ability - if they do not succeed in exploiting their accommodation properties - to behave as monofocal lenses, which (given the absence of an important astigmatism and a biometric IOL calculation performed in a workmanlike manner) often provide good natural vision for far distances [12–14, 39]. In binocular vision, in fact, the FIL618 lenses have shown to induce a negligible myopia in the sample under evaluation in this study, amounting to a spherical equivalent of 0.23 D with a natural logMAR visual acuity for distance of 0.14, which isn’t improved in a statistically significant way with the usage of spectacles.

Patients that received FIL618 accommodative lenses referred a humble visual acuity for close-up distances, thus requiring almost constantly an additional correction to obtain the best visual. All the findings were in accordance with what stated by the manufacturer (which reports a potential additional correction of +2.00 D for FIL 618 - if considered at the plane of spectacle lenses) and with what reported by patients in the satisfaction questionnaires, from which emerges a significant spectacle dependence of the patients for close distances. In this group, a larger capsulorhexis (minimum diameter 6 mm) has been performed, in order to facilitate movements of the IOL optic plate, along the anteroposterior axis, hence allowing a better accommodation ability. This theoretical assumption has not been confirmed by the correlation between capsulorhexis diameter - measured at the slit lamp - and additional correction requested postoperatively.

In agreement with the data of the literature, halos and glare perceptions are a quite frequently feature associated with the use of multifocal lenses, either refractive or diffractive [40]. SN6AD1 patients referred rarer halos and glare, with better performances in night vision, too.

The difficulty in reading small fonts reported in literature for the AMO Tecnis multifocal lenses and AcriTec TwinSet [41–45] was not been confirmed by our lenses FIL611PV, which much like the Restor SN6AD1, proved very promising performance in natural vision from intermediate/close, also in monocular vision [46–50].

Concerning intermediate distance, in agreement with the results of literature [51, 52], multifocal lenses FIL611PV have met the expectations and needs of our patients, with the advantage of greater spectacle independence.

## Conclusion

In agreement with the literature data, multifocal lenses tested in this study are a viable solution for visual rehabilitation after cataract extraction surgery performed with the intent to make phacoemulsification and IOL implantation real refractive measures in order to improve as much as possible patient autonomy for different working distances. These assumptions are valid depending by the choice of the lens that best suits patient’s visual demands. If in fact the Restor SN6AD1 lenses seem to guarantee the best visual performance in all working distances, FIL611PV may represent a promising alternative at a considerably cheaper cost, more oriented to prioritize vision for intermediate/close-up distance, which may lead to greater autonomy especially in elder subjects.

Our study revealed that FIL618 accommodative IOLs seem to provide decent distance visual acuity, especially in binocular conditions, in contrast with what stated by the current literature (which reports greater discomfort in far distances vision for accommodative lenses and minimum for multifocal lenses) [13, 14, 17–19, 29, 53]. FIL618 lenses appear to be well tolerated in terms of halos and “glare” perception, which, in agreement with literature data, are a feature that is rather frequently associated with the use of multifocal lenses, of both refractive and diffractive type [6, 17, 18, 54]. However, the observed FIL618 performance in terms of UCNVA was poorer, probably due to an accommodation potential limited to only +2 diopters, resulting in lower spectacle independence in close-up vision and tasks. Future perspectives for accommodative IOLs applications lie in the manufacturing of lenses with greater accommodation amplitude (of at least +3 D), which are not currently available.

From all evaluations brought to light by our study, although small and worthy of further development, emerges the great importance of taking into consideration different features of different IOLs models. In fact, it is advisable to have great care of patients demands and needs while carrying out the choice of the right IOL that may lead to best satisfaction and autonomy in performing daily tasks at given working distances. It is important to consider each different IOL characteristics and performances even when modifications of post-operative astigmatism are intended. Finally, AcrySof ReSTOR IOLs, despite their excellent performances, (especially for the SN6AD1 model) have the disadvantage of being considerably more expensive in comparison with FIL611PV. Further knowledge of IOLs features and different surgical arrangements may lead to the development of a more personalized and refractive-oriented cataract surgery, allowing also patient’s visual rehabilitation.
